# The Expression Level of mRNA, Protein, and DNA Methylation Status of* FOSL2* of Uyghur in XinJiang in Type 2 Diabetes

**DOI:** 10.1155/2016/5957404

**Published:** 2016-12-06

**Authors:** Jun Li, Siyuan Li, Ying Hu, Guolei Cao, Siyao Wang, Partab Rai, Xiaoli Wang, Kan Sun

**Affiliations:** ^1^Department of Endocrinology and Metabolism, The First Affiliated Hospital, Shihezi University School of Medicine, Shihezi, Xinjiang 832002, China; ^2^Medical College, Shihezi University, Shihezi 832002, China; ^3^The First Department of General Medicine, The Affiliated Tumor Hospital of Xinjiang Medical University, Urumqi, Xinjiang 830000, China; ^4^Department of Endocrinology and Metabolism, The Fifth Affiliated Hospital, Xinjiang Medicine University, Urumqi, Xinjiang 830000, China

## Abstract

*Objective.* We investigated the expression levels of both* FOSL2* mRNA and protein as well as evaluating DNA methylation in the blood of type 2 diabetes mellitus (T2DM) Uyghur patients from Xinjiang. This study also evaluated whether* FOSL2* gene expression had demonstrated any associations with clinical and biochemical indicators of T2DM.* Methods.* One hundred Uyghur subjects where divided into two groups, T2DM and nonimpaired glucose tolerance (NGT) groups. DNA methylation of* FOSL2* was also analyzed by* MassARRAY* Spectrometry and methylation data of individual units were generated by the EpiTyper v1.0.5 software. The expression levels of* FOS-like antigen 2 (FOSL2)* and the protein expression levels were analyzed.* Results.* Significant differences were observed in mRNA and protein levels when compared with the NGT group, while methylation rates of eight CpG units within the* FOSL2* gene were higher in the T2DM group. Methylation of CpG sites was found to inversely correlate with expression of other markers.* Conclusions.* Results show that a correlation between mRNA, protein, and DNA methylation of* FOSL2* gene exists among T2DM patients from Uyghur.* FOSL2* protein and mRNA were downregulated and the DNA became hypermethylated, all of which may be involved in T2DM pathogenesis in this population.

## 1. Introduction

The incidence of diabetes mellitus (DM) and prediabetes mellitus is estimated at approximately 113.9 million and 493.4 million people, respectively [1]. In Xinjiang, there are no less than 13 ethnic groups with Uyghurs accounting for 46% of the total population. The prevalence of T2DM among this group is estimated at about 6.23% [2], and those considered prediabetic account for 31.6% of the population [3]. Xie et al. found that Uyghurs with type 2 DM (T2DM) had a higher body mass index (BMI) [4]. Chronic complications of T2DM, including heart and cerebrovascular disease, visual impairment, and end-stage renal disease, can contribute to high morbidity and mortality, which also creates economic burdens within society and families.

Studies have confirmed that many human diseases are associated with DNA methylation. During DNA methylation, methyl groups are added to CpG sequences within the promoter region [5]. Previous studies have shown that DNA methylation can be changed by age and life style, which suggests that this may be involved in metabolic diseases including T2DM [6–12].

The* FOS-like antigen 2 (FOSL2)* gene is located on chromosome 2 and has a full length of 21.74 kB. The* Fos* gene family is constituted by* FOSL2, FOS, FOSB,* and* FOSI*, and* FOSL2* has been described in many different tissue types in both animals and humans [13]. Recent studies have found that it plays a crucial role in forming fat cells; meanwhile, some researchers have demonstrated that metabolism can be affected by* FOSL2* expression [14, 15].

However, the pathogenic potential of methylated* FOSL2* in T2DM still remains unknown, especially in the Uyghurs of Xinjiang. Our study aimed to observe the methylation levels of* FOSL2* and analyze its correlation with clinical and biochemical indicators of T2DM within this population.

## 2. Materials and Methods

### 2.1. Research Subjects Samples

Participants were recruited from Uyghur patients in Xinjiang from September 2013 to March 2014. The subjects were divided into the T2DM (*n* = 50) and nonimpaired glucose tolerance (NGT; *n* = 50, as controls) group; because of the remoteness of the region, glycemic control within the blood samples is difficult.

All patients underwent a standard 75 g oral glucose tolerance test. Diagnosing T2DM is based on World Health Organization criteria. Exclusion criteria for the NGT group included T2DM and impaired glucose tolerance. Patients diagnosed with type 1 diabetes or autoimmune diseases or suspected severe heart disease, liver disease, kidney disease, or malignant tumor were excluded. The clinical characteristics of the subjects are listed in Table 1. Metabolic markers including fasting plasma glucose (FPG) and total cholesterol (TC) were measured using clinical chemistry analyzer. Fasting insulin (FINS) was measured by automatic chemiluminescence analyzer. Hemoglobin A1c (HbAlc) was measured by high pressure liquid chromatography. BMI was defined as one's weight in kilograms divided by the square of one's height in meters. Waist hip ratio was calculated using the waist hip circumference ratio. The insulin resistance index (HOMA-IR) and pancreatic *β* cell function index (HOMA-*β*) were calculated and analyzed using the Homeostasis Model Assessment (HOMA).

All participants signed informed consent at the beginning of the study. This study strictly followed the principles of the Declaration of Helsinki and was approved by the Ethics Committee of the Shihezi University, Xinjiang, China.

### 2.2. Nucleic Acid Isolation in Leukocyte and Protein in Serum

Total RNA was extracted with the RNAprep Pure Blood Kit (Tiangen Biotech, Beijing, China). DNA was extracted from blood cells using DNeasy Blood and Tissue Kit (Qiagen, Germany) according to manufacturer's instruction, and the productivity and purity of DNA were measured using a NanoDrop spectrophotometer. The nucleic acid samples were quantified by measuring their absorption at 260 nm. Protein in serum was measured by* FOSL2* enzyme-linked immunosorbent assay kit (ELISA, R&D Systems, MN, USA); the termination reaction results in photometric color change from blue to yellow.

### 2.3. Reverse Transcription-Polymerase Chain Reaction (RT-PCR)

RevertAid™ First Strand cDNA Synthesis Kit (Qiagen) was used. We combined the reverse transcription of RNA and polymerase chain reaction of cDNA to measure the mRNA expression of* FOSL2*. The primers for* FOSL2* were designed by EpiDesigner website (http://www.epidesigner.com/) as follows: forward 5′-CCAGATGAAATGTCATGG C-3′ and reverse 5′-CTCGGTTTGGTAGACTTGGA-3′ and the primers for*β-actin* as follows: forward 5′-CCCAGCACAATGAAGATCAAGATCAT-3′ and reverse 5′-ATCTGCTGGAAGGTGGACAGCG-3′. The reaction conditions for the PCR were as follows: 94°C for 4 min then 72°C for 2 min, followed by 36 cycles at 94°C for 30 sec, 56°C for 30 sec and 72°C for 30 sec. The level of mRNA was analyzed by a gel imaging system (Bio-Rad Gel Doc 2000, USA).

### 2.4. Methylation of* FOSL2* Gene

DNA was modified by sodium bisulfite with the reaction condition as follows: 20 cycles at 95°C for 30 sec and 50°C for 15 min. The sequence of the* FOSL2* gene was queried from University of California Santa Cruz genome biological information network (http://genome.ucsc.edu/). The primers for* FOSL2* were designed by the EpiDesigner website as follows: forward 5′-AGGAAGAGAGGTAGGTTTAGGA GAGGGGTGTG-3′ and reverse 5′-CAGTAATACGACTCACTATAGGGAGAAGG CTACAACCCCCAAAACTTAACTAAAAC-3′, primers for the CpG island of* FOSL2* were used to amply bisulfite treated DNA. The reaction conditions for the PCR were as follows: 94°C for 4 min and then 72°C for 3 min, followed by 45 cycles at 94°C for 20 sec, 56°C for 30 sec, and 72°C for 1 min. The PCR products were spotted on a 384-pad SpectroCHIP (Sequenom, San Diego, CA, USA), followed by spectral acquisition on a MassARRAY Analyzer. Methylation data of individual units were generated by the EpiTyper v1.0.5 software (Sequenom).

### 2.5. Statistical Analysis

The data were analyzed by SPSS17.0 statistical software and correspond to a normal distribution. Data are presented as mean ± standard error. Student's *t*-test was used to compare the mean of two independent groups. Correlation analysis was used between different indicators. Regression analysis was used in the relationship between variables. *P* < 0.05 was the cut-off for statistical significance.

## 3. Results

### 3.1. Clinical and Metabolic Characteristics of Subjects

Fifty Uyghur patients with T2DM and 50 healthy Uyghur participants in the NGT group were used in this study. The clinical and metabolic anthropometric characteristics of subjects involved in the study are showed in Table 1. Compared with NGT group, patients with T2DM showed higher BMI, FPG, HbA1c, TC, triglyceride (TG), low density lipoprotein cholesterol (LDL-C), and FINS levels when compared to control, while T2DM group showed lower high density lipoprotein cholesterol (HDL-C), insulin sensitivity index (ISI), and HOMA-*β* which is function index of *β*-cell and index of insulin resistance (IR) levels.

### 3.2. Comparison of the Expression of* FOSL2* mRNA between NGT and T2DM Groups in Uyghurs in Xinjiang

**Figure 1:**
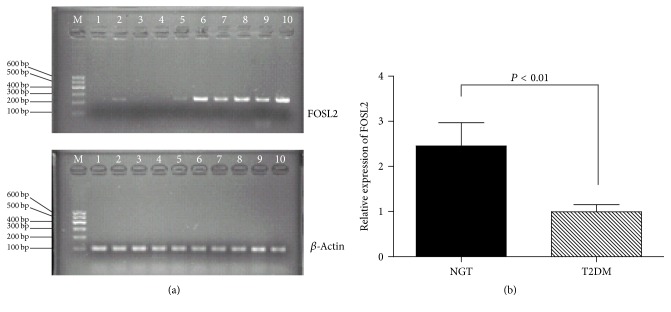
The comparison of the expression of* FOSL2* mRNA between the NGT and T2DM groups in Uyghurs in Xinjiang. (a) Electrophoretogram of* FOSL2* mRNA. M: molecular weight marker; *β*-actin was the internal reference gene. 1–5 and 6–10 in (a) were the* FOSL2* of T2DM and NGT groups, respectively, in Uyghurs in Xinjiang. (b) The mRNA relative quantification expression of* FOSL2* in the two groups.

To compare the expression of* FOSL2*, we measured the mRNA in the NGT and T2DM groups. The results showed that the gene expression level of* FOSL2* in the T2DM group was significantly lower than that of the NGT group (Figure 1(a)). Further we did relative quantification analysis in the two groups, which revealed the same trend (2.46 ± 0.51, 0.10 ± 0.16; *P* < 0.01) (Figure 1(b)).

### 3.3. Comparison of the Expression Levels of* FOLS2* Protein

**Figure 2:**
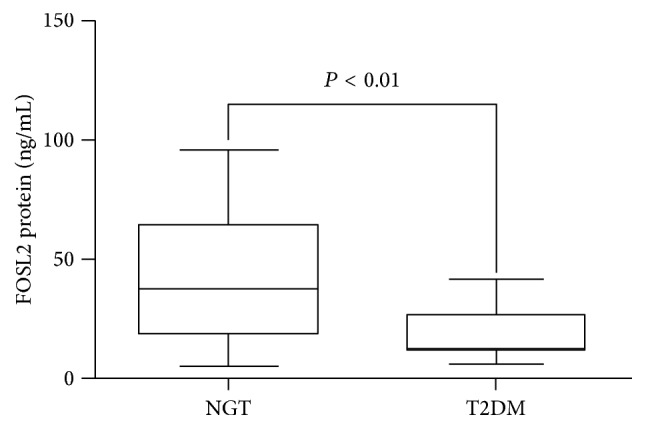
The comparison of the expression levels of* FOSL2* protein between the NGT and T2DM groups. Protein was measured by ELISA. Data are presented as the mean ± standard deviation (SD).

Compared with NGT group, the T2DM group had a significantly lower protein expression level for* FOSL2* (41.48 ± 26.32 ng/mL, 18.09 ± 9.48 ng/mL, *P* < 0.01) (Figure 2).

### 3.4. Comparison of the Mean Methylation Level of* FOSL2* in Two Groups

**Figure 3:**
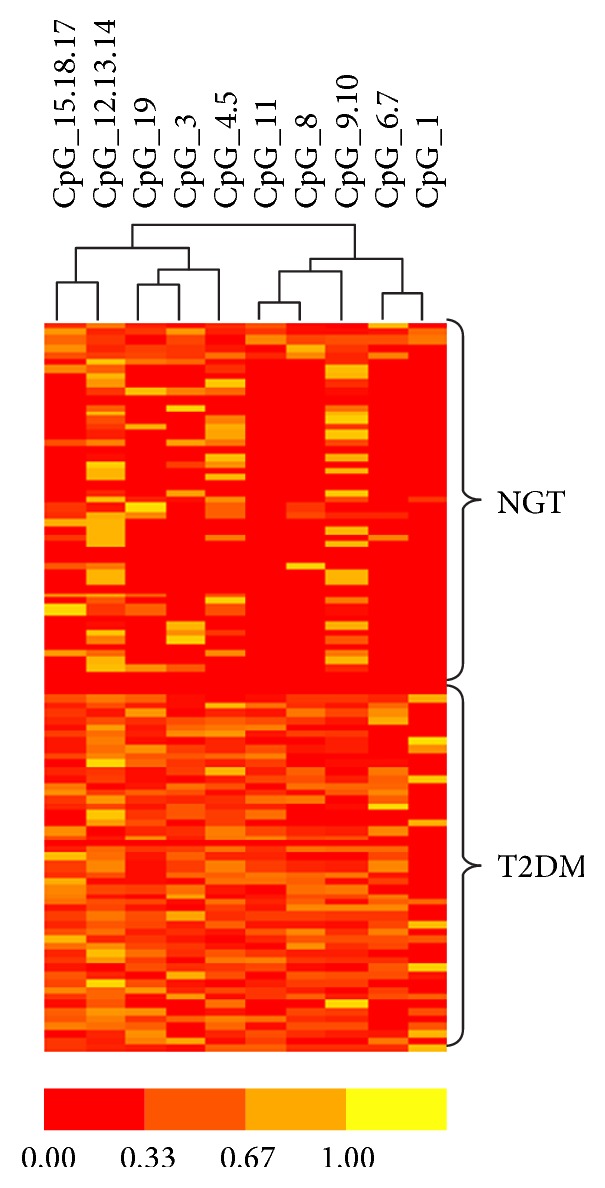
Hierarchical clustering of* FOSL2* methylation between NGT and T2DM groups was measured by MassARRAY analysis. Each row reveals a subject. Each column reveals a CpG unit. Color coding represents the methylation level; yellow presents 100% and red 0%.

**Figure 4:**
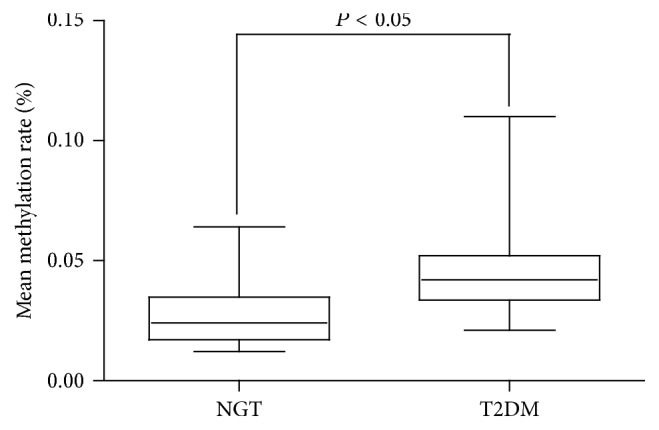
The DNA methylation levels of* FOSL2* in NGT and T2DM groups. The mean methylation levels in 100 samples between two groups were determined by MassARRAY and the data are presented as mean ± standard deviation (SD).

MassARRAY was used to analyze the data from 50 NGT and 50 T2DM samples. Cluster 3.0 was used to distinguish the differences between these two groups. The data generated by Cluster was analyzed by TreeView (Figure 3).* FOSL2* DNA methylation showed a significant difference with higher methylation in T2DM (0.03 ± 0.01, 0.05 ± 0.02; *P* < 0.05) (Figure 4).

### 3.5. Analysis Methylation Status of* FOSL2* Gene in the NGT and T2DM Groups

**Figure 5:**
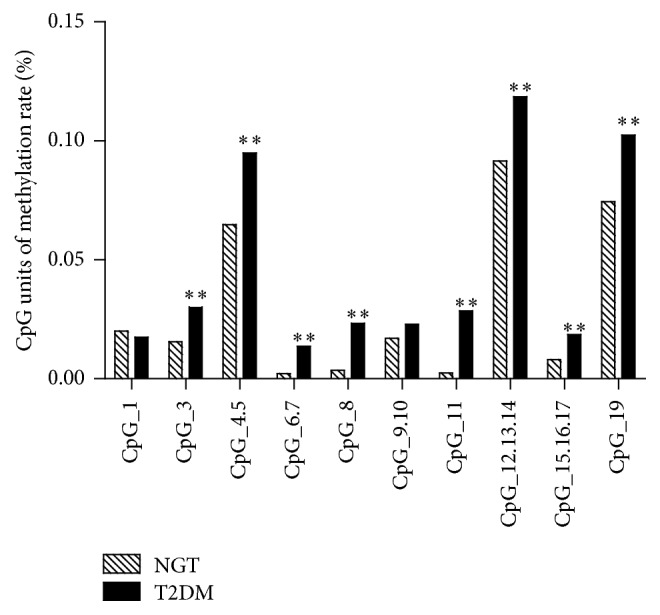
Analysis of the methylation status of* FOSL2* in the T2DM and NGT groups. DNA methylation changes of* FOSL2* in T2DM and NGT groups. Eight CpG units for DNA methylation in the* FOSL2* promoter showed higher methylation in T2DM group. ^*∗∗*^ *P* < 0.01.

The results of* FOSL2* gene methylation studies showed that the methylation rate of CpG units (CpG_3, CpG_4.5, CpG_6.7, CpG_8, CpG_11, CpG_12.13.14, CpG_15.16.17, and CpG_19) was higher in the T2DM group when compared to the NGT group (*P* < 0.01) (Figure 5).

### 3.6. Analysis of the Correlation between Methylation Level and mRNA of* FOSL2*

**Figure 6:**
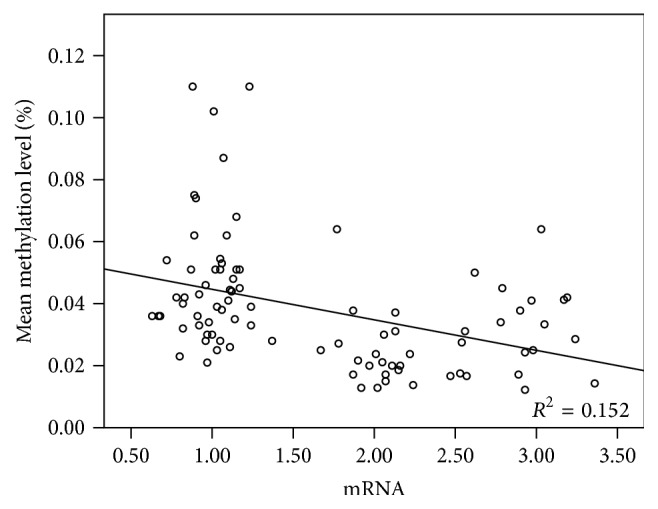
Analysis of the correlation between methylation level and mRNA expression of* FOSL2*. The methylation level was set as the *y*-axis and mRNA as the *x*-axis in regression analyses.

The correlation analyses showed a negative relationship with a correlation coefficient of −0.39 between methylation level and mRNA expression of* FOSL2*. The regression equation is as follows: *Y* = 0.54 − 0.01*X* (Figure 6).

### 3.7. Relationship Analysis between Methylation of CpG Units and Clinical Characteristics in Uyghur T2DM Patients in Xinjiang

The correlative analysis showed that methylation of CpG6.7, CpG8, CpG11, CpG12.13.14, and CpG15.16.17 was positively correlated with FPG; CpG11 and CpG12.13.14 were positively correlated with FINs; TC and TG were positively correlated with CpG12.13.14; CpG6.7, CpG8, CpG11, CpG12.13.14, and CpG15.16.17 were negatively correlated with ISI and methylation of CpG11 and CpG12.13.14 was negatively correlated with HOMA-*β* (Table 2).

## 4. Discussion

DM has become a major public health problem and as a result of its very high incidence and morbidity there is an urgent need for a sustainable public health solution. T2DM has especially high incidence among the Uyghur populations in Xinjiang. However, the specific mechanism of T2DM development remains unknown and to date,* FOSL2* gene methylation has not been investigated in T2DM patients.

Leptin plays important roles in regulating blood glucose and lipid metabolism and has been found to play a negative role in weight gain. It may have a profound impact on insulin resistance [16]. Wrann et al. found that* FOSL2* was positively correlated with* leptin* expression [17].

The* FOSL2* gene is widely expressed in various organs and tissues during both human and animal development [18]. The* Fos* gene family plays an important role in cell differentiation and proliferation [19]. Meanwhile,* FOSL2* gene expression has been linked to fat metabolism, cancer, and bone diseases in human and animals [14, 20–22].

Our main findings include* FOSL2* expression, at both the protein and mRNA levels, which is significantly reduced in the T2DM group when compared to the NGT group. This reveals a correlation between decreased* FOSL2* expression and T2DM, which reveals that the loss of* FOSL2* expression is correlated with T2DM. Secondly, 19 CpG sites were described and their methylation state was evaluated; we showed that there is a measured increase in methylation of these CpG islands in the T2DM. DNA methylation has been previously described in other genes associated with T2DM; Ling et al. found that hypermethylation may reduce the mRNA and secretion of insulin [23]. This association was also present in* FOSL2*. The increased methylation of* FOSL2* in the T2DM group correlates with the reduced mRNA levels described in the same group. This association mirrors the observations in the Ling et al. study. Furthermore, previous research from Liliand et al. indicated that DNA methylation might regulate blood lipid levels and lead to metabolic disease [24]. In our study, CpG sites were found inversely related to TC and TG levels. Subjects in the T2DM group also had higher blood lipid levels, providing evidence that the hypermethylation of* FOSL2* might be influenced by TC and TG in T2DM group. Brøns et al. found that blood lipid levels increased with DNA methylation levels and decreased insulin secretion and induced insulin resistance [25]. The correlative analysis in our study showed that methylation of the CpG sites was positively related to FPG and FINS, while negatively correlated with ISI and HOMA-*β*; these coincide with the Brøns et al. study. The correlation between the index of insulin resistance HOMA and methylation of repetitive sequences of* Alu* was also reported by Zhao et al. [26]. Our results are in line with this study, which showed that glucose and lipid metabolism disorder was the important cause of islet *β*-cell dysfunction and DNA methylation is significantly associated with insulin resistance [27], resulting in a greater risk of DM. The dysfunction of islet *β*-cell in our study supports the same conclusion. Our data shows that* FOSL2* which is related to glycol-metabolism and pancreas development is hypermethylated in subjects with T2DM. Also, we propose that the high blood glucose and high plasma lipid levels may be positively correlated with hypermethylation and the dysfunction of islet *β*-cells is a result of hypermethylation. One of the limitations in this study is the choice of experimental method; real-time quantitative PCR may be a better choice for validation in the future. A second, more problematic limitation is the low sample size, and thus our results should be verified in a larger cohort.

In conclusion,* FOSL2* in T2DM patients was hypermethylated and exhibited lower mRNA and protein expression levels. The results suggested that DNA methylation may contribute to these lower expression levels, which may in turn contribute to the onset of T2DM. Our data supports the hypothesis that methylation may be an early event in the development of diabetes [28]. These observations provide evidence that* FOSL2* methylation state may be a useful biomarker for increasing risk of T2DM progression within the Uyghur population in Xinjiang and preventing a potential novel target for therapeutic development. T2DM often leads to abnormal bone mineral density; Mathen et al. found that subjects with T2DM had lower bone mineral density [29]. Longitudinal studies are needed to clarify the methylation state of* FOSL2* among osteoporosis and T2DM with osteoporosis patients; it is worthwhile to investigate whether* FOSL2* promotes the formation of osteoblasts and the role of* FOSL2* in this and other metabolic diseases.

## Figures and Tables

**Table 1:** Clinical and metabolic characteristics of Uyghurs between NGT and T2DM groups.

Parameter	NGT group	T2DM group
*n*	50	50
Gender (male/female)	26/24	22/28
Age (years)	51.58 ± 13.51	54.46 ± 9.43
SBP (mmHg)	131.20 ± 22.46	134.10 ± 17.92
DBP (mmHg)	83.70 ± 15.35	86.98 ± 14.73
WHR	0.94 ± 0.09	0.97 ± 0.06
BMI (kg/m^2^)	25.02 ± 3.19	28.43 ± 4.01^*∗∗*^
FPG (mmol/L)	5.45 ± 0.69	11.40 ± 4.39^*∗∗*^
HbAlc (%)	6.21 ± 1.30	10.20 ± 2.10^*∗∗*^
TC (mmol/L)	3.80 ± 0.87	5.16 ± 0.87^*∗∗*^
TG (mmol/L)	1.48 ± 0.85	2.21 ± 1.02^*∗∗*^
HDL-C (mmol/L)	1.19 ± 0.30	1.03 ± 0.34^*∗*^
LDL-C (mmol/L)	2.26 ± 0.83	2.63 ± 0.74^*∗*^
FINS (mIU/L)	11.61 ± 0.94	16.13 ± 1.61^*∗*^
ISI	−3.90 ± 0.80	−4.93 ± 0.73^*∗∗*^
HOMA-*β*	4.65 ± 0.85	3.63 ± 0.97^*∗∗*^
IR	2.74 ± 1.59	7.81 ± 5.32^*∗∗*^

SBP, systolic blood pressure; DBP, diastolic blood pressure; WHR, waist hip radio; BMI, body mass index; FPG: fasting plasma glucose; HbA1c: hemoglobin Alc; TC: total cholesterol; TG: triglyceride; HDL-C: high density lipoprotein cholesterol; LDL-C: low density lipoprotein cholesterol; FINS, fasting plasma insulin; ISI, insulin sensitivity index; HOMA-*β*: function index of *β*-cell; IR: index of insulin resistance. *P* value: *∗* < 0.05, *∗∗* < 0.01.

**Table 2:** Correlation between methylation units and clinical characteristics.

CpG unit	WHR	FPG	IR	ISI	HOMA-*β*	TC	TG
CpG6.7	0.064	0.229^*∗*^	0.178	−0.261^*∗*^	−0.174	0.095	0.153
CpG8	0.134	0.253^*∗*^	0.200	−0.298^*∗∗*^	−0.189	0.141	0.154
CpG11	0.077	0.330^*∗∗*^	0.225^*∗*^	−0.336^*∗∗*^	−0.276^*∗*^	0.201	0.166
CpG12.13.14	0.007	0.241^*∗*^	0.212^*∗*^	−0.254^*∗*^	−0.240^*∗*^	0.284^*∗∗*^	0.251^*∗*^
CpG15.16.17	0.063	0.226^*∗*^	0.180	−0.235^*∗*^	−0.150	0.123	0.092
CpG19	0.452^*∗∗*^	0.113	0.189	−0.187	−0.096	−0.081	0.125

*∗* < 0.05, *∗∗* < 0.01. WHR, waist-hip ratio; FPG, fasting plasma glucose; IR, insulin resistance index; ISI, insulin sensitivity; HOMA-*β*, homeostasis model assessment of *β*-cell function index. TC, total cholesterol; TG, triglycerides.
